# Iterative pseudo balancing for stem cell microscopy image classification

**DOI:** 10.1038/s41598-024-54993-y

**Published:** 2024-02-24

**Authors:** Adam Witmer, Bir Bhanu

**Affiliations:** 1 Department of Bioengineering, University of California, Riverside, CA 92521 USA; 2 Department of Electrical and Computer Engineering, University of California, Riverside, CA 92521 USA

**Keywords:** Deep learning, Stem cell microscopy, Pseudo-labels, Stem-cell biotechnology, Huntington's disease, Cellular imaging, Pluripotent stem cells, Image processing, Machine learning, Stem-cell biotechnology, Huntington's disease, Cellular imaging, Pluripotent stem cells, Image processing, Machine learning

## Abstract

Many critical issues arise when training deep neural networks using limited biological datasets. These include overfitting, exploding/vanishing gradients and other inefficiencies which are exacerbated by class imbalances and can affect the overall accuracy of a model. There is a need to develop semi-supervised models that can reduce the need for large, balanced, manually annotated datasets so that researchers can easily employ neural networks for experimental analysis. In this work, *Iterative Pseudo Balancing (IPB)* is introduced to classify stem cell microscopy images while performing on the fly dataset balancing using a student-teacher meta-pseudo-label framework. In addition, multi-scale patches of multi-label images are incorporated into the network training to provide previously inaccessible image features with both local and global information for effective and efficient learning. The combination of these inputs is shown to increase the classification accuracy of the proposed deep neural network by 3$\%$ over baseline, which is determined to be statistically significant. This work represents a novel use of pseudo-labeling for data limited settings, which are common in biological image datasets, and highlights the importance of the exhaustive use of available image features for improving performance of semi-supervised networks. The proposed methods can be used to reduce the need for expensive manual dataset annotation and in turn accelerate the pace of scientific research involving non-invasive cellular imaging.

## Introduction

Stem cell biology is a promising field of study that has applications in the areas of regenerative medicine and disease modeling^1,2^. In-vitro experimentation is paramount to determining the underlying mechanisms of cellular growth and differentiation that contribute to a more comprehensive understanding of stem cell biology. However, one bottleneck in experimentation is the reliable and robust analysis of non-invasive microscopy imaging used to observe cellular changes. This work proposes to use deep learning algorithms to automatically analyze microscopy images containing multiple cell types with dynamic morphological structure. Specifically, problems arising from highly imbalanced datasets are addressed here using pseudo-labeling to allow for the use of small, imbalanced datasets for training data-expensive neural networks to characterize stem cell health and differentiation status. The models presented in this work will allow for more efficacious implementation of deep learning in research based settings to reveal key insights into stem cell biology for use in a variety of applications.

Stem cell differentiation is a delicate in-vivo process that orchestrates embryonic changes from naive pluripotency to somatic lineage commitment in all living organisms^3^. These cells are most susceptible to harm at the earliest stages of growth and development, when the small number of cells that make up the embryo are easily affected by environmental toxicants and mutagens^4^. The biochemical understanding of the underlying mechanisms of these processes has been harnessed towards technologies that are aimed at regenerative medicine and cellular manipulation such as induced pluripotent stem cells (iPSC), which are a useful tool for researchers to understand the developmental differentiation process including models of disease^5,6^.

One such disease, Huntington’s disease (HD), is a neuro-degenerative disorder that affects the HTT gene in the human brain^7^. This mutation causes a repeat expansion of the CAG codon at the terminus of the Huntingtin protein. This protein is instrumental in neuronal maturation and migration, and the functional gain incurred by HD expression prompts these processes to occur at a much higher rate. Like other proteopathic disorders (e.g. Parkinson’s, ALS, etc.), HD manifests itself in muscular skeletal disfunction and rapid cognitive decline^8–10^. These symptoms display themselves at later stages of life, and there is evidence to suggest that nicotine has a neuroprotective effect that can slow disease progression^11,12^. Given this, and the fact that HD has been shown to affect human neurodevelopment in both humans and mice by causing neural progenitor cells to mature at a faster rate, it may indicate that nicotine could have the same effect on developing stem cells^8^.

Live cell microscopy of stem cell disease models is crucial to the research efforts that have made these important discoveries. These non-invasive methods allow scientists to observe cellular behavior in response to experimental stimuli, and predict outcomes based solely on morphological patterns. Video-bioinformatics (VBI), which is defined as “the automated processing, analysis, understanding, data mining, visualization, query-based retrieval/storage of biological spatiotemporal events/data and knowledge extracted from videos obtained with spatial resolution varying from nanometer to meter of scale and temporal resolution varying from seconds to days and months,” has led to the advancement of programs aimed at quantification of stem cell events^13^. Such programs include those that use traditional computer vision, pattern recognition, and machine learning classification techniques^14–16^. Others have recently adopted deep learning, which combines the feature extraction and classification modules, learns a more robust representation of input images and significantly out-performs hand-designed methods in a majority of VBI tasks^17–21^.

The unique circumstances of biological experimentation and data collection (i.e., cost, time-constraints, uncertainty in experimental outcomes, and lack of image data) reveal some drawbacks to deep learning. Factors such as class imbalances, contiguous boundaries, multi-label images, anomalies, lack of data, and the prerequisite of manual annotations hinder the ability of researchers to easily employ deep learning models for their analytical pipeline. These limitations are a major hurdle in training neural networks using biological images.

For example, in the case of stem cell microscopy, many multi-label images are collected that contain more than one morphological class in a single image. It is very difficult to use multi-label images for supervised training because they cannot be accurately annotated without invasive biomarker validation. Unlike natural image datasets, the task of biological image annotation must be performed by a domain expert or someone trained to specifically analyze a particular dataset. Even with this requirement satisfied, reliable annotations cannot always be guaranteed given the high degree of variability within biological images.

Even when manual annotations have been provided to every image, a subsequent problem is the innate imbalances in the dataset class distribution. In stem cell biology, these imbalances arise from the pluripotency of cells before undergoing differentiation, meaning that they have the ability to change into any mature cell type. In-vivo, these changes are orchestrated by a very specific and well-defined cascade of signals from the developing embryo^22^. However, in-vitro they are subject to a measure of uncertainty that results in an imbalance in proportions of the observed image class samples^23^. This randomness, coupled with the downstream differentiation of cells towards somatic lineages causes variation in the number of cells at any given stage of development during the experimental growth cycle.

These drawbacks can be overcome by leveraging the power of semi-supervised learning algorithms rather than those trained end-to-end in a fully supervised manner. These algorithms allow the network to estimate features from an imbalanced, unlabeled dataset and fine-tune these features via reinforcement from a smaller, balanced, labeled subset. In a practical setting, this allows researchers to hand label a smaller portion of their dataset and train a network on both labeled and unlabeled data without having to perform extensive manual annotations. This saves time and resources and improves analytical workflows involving deep learning.

Therefore, this paper introduces Iterative Pseudo Balancing (IPB), which uses meta-pseudo-labeling to balance a dataset of biological images on-the-fly during model training and improve the performance of a deep neural network for the task of stem cell colony classification. The outline of the paper is as follows: First, related works are discussed and compared to the proposed method, and the unique contributions of the paper are clearly stated along with their significance. Second, an overview of the technical approach is presented, along with in-depth explanation of the novel aspects of the network architecture, algorithm, and experimental configurations. Finally, the results of these experiments are summarized and interpreted, and inferences are drawn as to the reasons behind the observed patterns. Ultimately, it is determined that the method presented in this work improves over previous works by exploiting domain knowledge for network training, and advances the state-of-the-art by solving the problem of class imbalances in biological image datasets.

### Related work and contributions

Semi-supervised deep learning has recently been leveraged for image classification tasks with a popular method being the contrastive learning networks. Contrastive learning refers to training a neural network by comparing similar and dissimilar image samples and updating the feature map in relation to a comparative loss function^24^. For example, SimCLR, proposed by Chen et al.^25^ uses contrastive learning to map input features based on the similarities and differences between individual images. At each iteration, a single image is considered a positive instance while all other images are considered negative instances. The network learns a general view of the input features, and then it is refined via supervised fine-tuning to incorporate class information. This is an effective self-supervised method, however, there is no way to account for class imbalances in contrastive learning without prior information, which limits the ability of the network to learn unbiased features. These algorithms also require an extremely large dataset for effective learning, which is counterproductive for a task involving limited datasets.

Another work by Chuang et al.^26^ called Debiased Contrastive Learning (DCL) attempts to improve upon the contrastive scheme by removing some randomness from the sample selection process. DCL compensates for sampling bias by estimating the probability distributions for the negative and positive classes. However, they assume that these distributions are uniform, which is often not the case in real world, biological settings.

Similarly, Li et al.^27^ introduce Contrastive Clustering, which uses two output layers to extract features at the instance and cluster levels. They try to approximate the feature space more closely by updating the network with respect to a joint contrastive loss function that combinies these two feature modules. They outperform various state-of-the-art clustering methods, but report that their method is highly dependent on data bias and more research is needed to improve model robustness to the level of real-world applications such as health care.

Previous studies have attempted to solve the problems associated with biological datasets, including limited data and imbalanced class distributions, using semi-supervised, self-supervised and contrastive deep learning methods. One such work by Murphy et. al.^28^ uses a SimCLR model to extract feature embeddings from a dataset of immunohistochemicstry (IHC) images and combine them with protein data to predict image biomarkers in a self-supervised manner. They implement a hard negative sampling scheme to counterbalance inherent dataset biases and outperform baseline methods of transcriptomic classification but fail to beat fully supervised training methods aimed at histopathology^29^. They note that the variability within IHC images and between image samples aids in the augmentation schemes necessary for effective learning using contrastive algorithms and theorize that multi-scale inputs would help to improve their training. However, their method utilizes concurrent genetic information in the form of scRNA sequencing data, whereas the method in this work seeks to determine class features based only on morphological information from non-invasive live-cell imaging.

Another work by Liu et al.^30^ aims to classify histopathology images using a contrastive network that combines the SimCLR framework with a triplet-net configuration to simultaneously minimize intra-class variance and maximize interclass variance. Their model, SimTriplet, avoids performing negative image sampling by assuming that adjacent patches within a large scale image contain features from the same image class, while patches from other image regions contain pertinent negative context. Their model overcomes the computational expense of the SimCLR model to improve overall accuracy for their classification task, however, they perform pre-balancing of the dataset to compensate for dataset imbalances, and only use a single-scale, down-sized image patch to improve training efficiency, as opposed to a multi-scale input.

Other similar patch based methods exist such as Visual Transformers (ViT), which break images down into patches to perform attention with state-of-the-art classification results, but require large datasets for effective learning (15-100M images)^31^. This requirement has a similar pitfall to contrastive learning, and can affect the results of networks trained on smaller datasets. A network called DeepFA, by Benato et al.^32^, attempts to solve this problem by using a student-teacher network that combines a 2D semi-supervised forest classifier with a meta-pseudo-label (MPL) module to learn image features from a small data subset on a variety of datasets. They use their method to solve the problem of low data availability, but use random sampling in their training and note dataset imbalances as a limitation of their work.

Given the drawbacks of the previously described methods, the approach outlined in this work aims to balance and classify a stem cell microscopy dataset using a semi-supervised student-teacher framework with multi-scale inputs from multi-label images. To the best of our knowledge, no other similar works have been published that address biological image classification using meta-pseudo-labeling. The specific contributions of this paper are as follows:

- Introduces Iterative Pseudo Balancing to classify stem cell microscopy images using semi-supervised learning and address dataset limitations surrounding manual annotation and class imbalances; improvements over state-of-the-art will allow researchers to accelerate their experimental analysis using deep learning without the need for large labeled datasets
- Performs on-the-fly dataset balancing via pseudo-label-resampling at the image patch level. This is the first use of psuedo-labeling for dataset balancing, which allows for neural network training with unlabeled datasets. The previous MPL algorithm^33^, did not account for imbalanced datasets, which is detrimental to model learning
- Incorporates previously inaccessible image features using patches of multi-label images to provide local and global features via multi-scale inputs. It is shown here empirically that the combination of these features is necessary for the greatest improvement in classification accuracy and improves over the single scale inputs of state-of-the-art related methods

## Technical approach

### Iterative pseudo balancing framework

Biological datasets present unique circumstances for image feature modeling and classification. Unlike the standardized natural image benchmark datasets such as ImageNet^34^ or CIFAR10^35^, medical and biological images often require extensive curation and pre-processing as well as special considerations for network architecture and training procedures. For example, regions-of-interest within microscopy images generally contain high variability in terms of local entropy and fine-grained patterns. Furthermore, manual annotation across an entire dataset for use in neural network training poses practical issues in terms of time, and analytical redundancy caused by having to pre-sort every image in the dataset before training the neural network. In other words, it defeats the purpose of training a machine learning algorithm for biological image classification if manual annotation must be performed on a large dataset to train, test, and validate the initial model.

**Figure 1:**
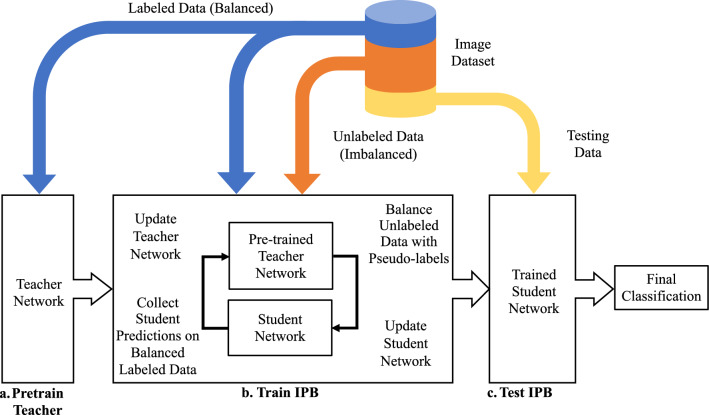
The overall diagram for the Iterative Pseudo-Balancing framework. (Top row) The dataset is divided into three parts, two smaller labeled subsets for training the teacher and testing the student, and a larger, unlabeled dataset for training the student network using iterative pseudo-balancing. (Bottom row) a. the teacher network is pre-trained on the balanced, labeled dataset. b. the IPB algorithm uses the pseudo-labels from the pre-trained teacher to resample and balance the unlabeled dataset during each epoch for training the student network. The teacher is then updated in relation to the classification performance of the students predictions on the labeled data. This process is repeated until the network converges. c. The trained student network is validated on the testing dataset. Details of this procedure are outlined in the section below.

**Figure 2:**
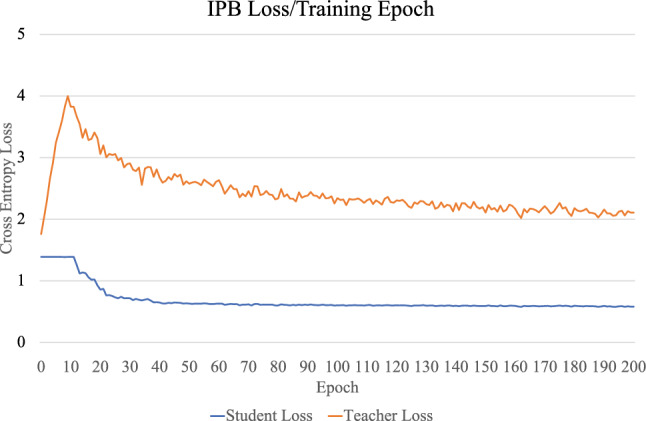
Training loss curves for the teacher and student networks, averaged over multiple runs. At the beginning of the training, the teacher loss increases while the student is still in the warm-up phase, where the learning rate is kept low to allow for the student to catch up to the teacher. After this phase, the teacher and student losses begin to go down, and stabilize over the course of training. As training progresses, the student network producing the best classification accuracy is taken as the network used for evaluation.

**Figure 3:**
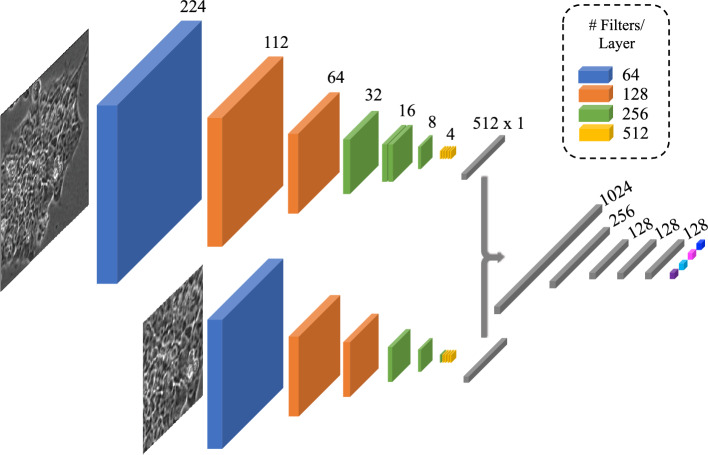
Overview of multi-scale VGG Network Architecture. Image patches of size 224 and 112 are provided as input to two separate network streams, from which feature vectors of 512 are concatenated and used to make a classification decision over the four classes. Numbers on top of features maps indicate image size at the corresponding cross section, and the legend in the top right displays the number of filter channels in each color coded layer. Batch normalization is added between every convolutional layer, as well as after the feature concatenation layer, and ReLU activation is used between all layers until the final classification.

Another very important consideration is the effect of class imbalances on semi-supervised learning. The meta-pseudo-labels^33^ algorithm utilized in this work suffers from overfitting as a result of confirmation bias when trained on imbalanced datasets. This causes the model to devalue the features of the least prevalent classes in its classification decision. Therefore, it is necessary to provide the semi-supervised model with a balanced view of the dataset classes to avoid overfitting on the most prevalent class. In this paper, Iterative Psuedo Balancing (IPB) is introduced to address these challenges by using semi-supervised pseudo-labeling to balance image classes. Figure 1 describes the proposed approach for the IPB framework. The numbered training and testing steps are as follows:

- Training Initialization
  1. The dataset is split by taking a portion of the images in each class for training the student using pseudo-labeled images (*U*), pre-training and updating the teacher network (*L*) and testing the student network (*T*).
  2. Training is performed over the course of *N* epochs, for which a single epoch is completed when the student network has seen every image in the unlabeled dataset (*U*).
  3. The HRNet configuration is used for both the teacher and student networks with the training specifications as shown in Fig. 3.
  4. The weights of both the teacher and student networks are initialized using Kaiming initialization^36^.
- Pre-train Teacher Network
  5. The teacher network is pre-trained, in a fully supervised manner, using a small, balanced, labeled subset of the dataset (*L*). Pre-training allows the teacher network to provide informed pseudo-labels to the unlabeled dataset during the iterative pseudo balancing phase, however, the teacher is also updated during the IPB phase in relation to the students performance on the labeled dataset.
- Train IPB
  6. The teacher network (initially pre-trained) is used to provide pseudo-labels to random image patches (one from each image) from the unlabeled dataset (*U*). The pseudo-labels are then used to balanced the imbalanced dataset using resampling from a multinomial distribution in relation to the weighted class proportions as determined by the teachers pseudo-labels.
  7. The balanced pseudo-labeled dataset is used to update the student network by collecting the students predictions on the pseudo-labeled images patches. The weights of the student network are updated in relation to the cross-entropy loss between the students predictions and the image pseudo-labels from the teacher (see Eq. (1)).
  8. To update the teacher, the predictions from the updated student network on the labeled images (*L*) are collected and the cross-entropy loss between the students predictions on the labeled dataset and the actual class labels is used to update the teacher (see Eq. (2)). This allows the teacher network to learn more robust pseudo-labels with each training epoch in order to provide the student with more accurate pseudo-labels.
  9. Steps (6-8), are repeated for *N* epochs until both the teacher and student networks converge, as monitored by the cross-entropy loss values from each network, as shown in Fig. 2. At the end of training, the student network with the highest classification accuracy over the course of training is taken as the final network for testing.
- Test IPB
  10. The trained student network is evaluated with the remaining testing data (*T*). One patch from each image in the testing dataset is provided to the network to perform image level testing. Network predictions for each image are collected and compared to the image labels for the testing dataset to perform final classification.
  11. The final classification is the maximum value of the softmax probability across the four morphological classes for every image in the testing dataset.

### Meta pseudo labels algorithm

As previously discussed, self-supervised and semi-supervised methods present a unique opportunity to avoid expensive pre-processing, curation, and manual annotation in training neural networks. However, these contrastive learning methods do not adequately account for dataset imbalances and fail to incorporate domain knowledge to guide model learning. Therefore, there is a need to address these issues using semi-supervised learning so that researchers can more effectively employ deep learning to standardize and accelerate their experimental analysis. To accomplish this, a meta-pseudo-labeling (MPL)^33^ algorithm is used as a basis for the proposed framework. MPL uses a student-teacher network configuration to learn features from unlabeled data, where a small amount of labeled data is used to first pre-train the teacher network, which then provides pseudo-labels for the other portion of unlabeled data at every iteration that is subsequently used to update the student network. The teacher network is updated in relation to the loss associated with classifying the labeled data using the student network as well as with a contrastive loss value inspired by Unsupervised Data Augmentation (UDA)^37^.

The loss functions for the teacher and student networks shown below highlight the relationship between the two networks during training. Equation (1) is the learning equation to update the parameters of the student network, where the updated student weights, ${\theta '}_S$, are calculated from the initial network weights, ${\theta }_S$ using the gradient ($\nabla _{\theta _S}$) of the cross-entropy loss, $\mathscr {L}_S$, between the pseudo-labels provided by the teacher network for the unlabeled images $\theta _T(x_u)$ (where $(x_u)$ is the unlabeled data) and the predictions of the student network on the pseudo-labeled images, $\theta _S(x_u)$, where $\eta _S$ is the learning rate of the student network. Equation (2) is the learning equation for updating the parameters of the the teacher network, ${\theta }_T$, using the gradient ($\nabla _{\theta _T}$) of the cross-entropy loss, $\mathscr {L}_l$, between the predictions of the updated student network on the labeled images, ${\theta '}_S(x_l)$, and the actual class labels, $x_l$, to obtain the new parameters of the teacher network ${\theta '}_T$, where the learning rate of the teacher is $\eta _T$. In this way, the teacher is iteratively learning from the student network and vice versa, such that the teacher can also learn more robust psuedo-labels for the unlabeled data.

1 $$\begin{aligned}{} & {} {\theta '}_S = \theta _S - \eta _S\nabla _{\theta _S}\mathscr {L}_S(\theta _T(x_u),\theta _S (x_u)) \end{aligned}$$

2 $$\begin{aligned}{} & {} {\theta '}_T = \theta _T - \eta _T\nabla _{\theta _T}\mathscr {L}_T(x_l, {\theta '}_S(x_l)). \end{aligned}$$

In a practical research setting, the MPL algorithm allows for a novel microscopy image dataset to be collected, partially annotated, and for the remainder of the raw data to be used as unlabeled input to train the student network in a semi-supervised manner. Consequently, this makes it possible to utilize patches of multi-label images in the training set without having to provide patch level semantic labels. For example, in the case of an experimental protocol for which multiple experimental folds are conducted, the researcher could use a single fold for training and testing the MPL network, and the other folds for cross-validation. In this scenario, the researcher could manually annotate a small portion of one fold for pre-training and testing the teacher network, use a larger, unlabeled, portion of that fold to train the student via MPL learning, and analyze the other folds using the trained network. Subsequent experiments with similar visual classes could then be analyzed in real time using the trained and tested student network without having to annotate more data, and network fine-tuning can be performed to incorporate new data classes. This setup overcomes the need for researchers to annotate the entire dataset for fully supervised learning, greatly improving the efficiency of the image analysis pipeline.

As previously stated the MPL algorithm does not inherently account for class imbalances within a dataset, which can lead to model bias and overfitting. Furthermore, none of the contrastive methods reviewed here include a multi-scale input, which limits the available features for the network to learn. Multi-scale inputs have been used for biological image classification to incorporate large scale image features while still allowing images to be classified by the fine-grained texture features of cellular image classes^38^. Therefore, the approach outline in this work, Iterative Pseudo Balancing (IPB), utilizes the pseudo-labels estimated by the MPL algorithm to iteratively resample a dataset of image patches such that for each epoch, the network is provided with a balanced dataset, helping to improve model learning. Furthermore, both multi-scale and multi-label inputs are used to improve feature extraction and classification of the network.

### Training procedures and architectures

Multiple network configurations are tested using the IPB algorithm to compare the effect of network architecture on stem cell classification accuracy. Different networks have certain advantages and drawbacks depending on the dataset because of the effect of receptive field on image feature mapping^39^. Specific parameter values such as kernel size, convolutional stride and overlap, and network depth and width contribute to the modeling of features and are often best suited for specific tasks. For example, VGG^40^ is optimal for modeling fine-grained features like those found in biological datasets, whereas ResNet^41^ is better for detection of larger objects and regions-of-interest (ROI). In this paper, the VGG19 architecture is used as a baseline configuration, and has been shown previously to produce superior results in comparison to ResNet on stem cell images^42^.

The High Resolution Network (HRNet)^43^ is also tested in this work as an example of a more recent network architecture that overcomes the loss of information in low level convolutions by combining network features in parallel to conserve high-level features in deeper layers. HRNet fuses layers along parallel branches in order to conserve spatial features at multiple resolutions, as opposed to convolutional networks that combine layers in series, which results in a loss of high-resolution information at deeper layers. In this way, HRNet is able to more effectively represent high and low level image features with a similar number of overall parameters and computational cost as other neural network architectures.

All network configurations are trained from scratch using 10-fold cross validation, with an 80:10:10, Unlabeled Training:Labeled Training:Testing dataset split. The 10% labeled data is chosen as a standard benchmark for semi-supervised learning methods and is used here to simulate a limited data setting^33^. The balanced, labeled training data subset, *L*, is used to pre-train the teacher network, as well as update the teacher during IPB training based on the students predictions over the labeled images. The unlabeled training data, *U* is used to train the student network with the pseudo-labeled patches from the pre-trained teacher. The labeled testing dataset, *T*, is used to evalutate the network at the end if IPB training, and is unseen by either of the networks during any of the training steps.

The HRNet used for both the teacher and student networks contains 10 convolutional layers and 5 fully connected layers for a total of 15 layers. All convolutional layers use 3$\times$3 filter kernels with stride of 1 pixels and padding of 1 pixel. Each convolutional layer is followed by a batch normalization function and ReLU activation. Down-sampling layers use 3$\times$3 filter kernels with stride of 2 pixels and padding of 1 pixel. The number of filter channels in each layer is given in Fig. 3. A batch size of 32 is used during pre-training and training of the IPB algorithm. Both the teacher and student networks are initialized using Kaiming Initialization^36^ before beginning any of the training steps.

Hyperparameters for the stochastic gradient descent optimizer were determined empirically and include a learning rate of 0.005, weight decay of 0.0001, and momentum of 0.9, as well as the number of training epochs, which is the epoch at which training stabilizes as determined by the cross entropy loss. The teacher network is pre-trained on the small labeled dataset for 200 epochs and the IPB network is trained for of 200 epochs, where one epoch is determined when the student sees every pseudo-labeled image of the unlabeled dataset. Dataset augmentations (described in the following section) are performed on the training dataset to increase image variability, and ensure spatial invariance. When IPB training is complete, the student network with the highest accuracy is taken for performing network evaluation using the labeled testing dataset. Figure 2 displays the loss function for the student and teacher networks. The IPB training algorithm performs 10 epochs for warm-up of the student network, as evident by the plateau present at the beginning of the loss curve for the student network.

### Data pre-processing

#### Cell colony detection

Before training, several data pre-processing steps are performed to reduce irrelevant information from input images. Firstly, the raw microscope images measure 2908 $\times$ 2908 pixels and contain several cellular colonies within a single image. To remove background area, these colony ROIs are extracted from the image using a morphological segmentation scheme that includes the following steps (with specific parameters and OpenCV (Version 4.5.5) functions included): 1. Gaussian blurring (cv2.GaussianBlur, kernel size 3$\times$3), 2. entropy filtering (skimage.filters.rank.entropy, disk filter size 3), 3. binarization via Otsu thresholding (skimage.filters.threshold_otsu), 4. morphological opening (skimage.morphology.opening, disk filter size 3), 5. hole filling (scipy.ndimage.morphology.binary_fill_holes), 6. small object removal (skimage.morphology.remove_small_objects with filter size of 2000 pixels).

Bounding boxes containing these binarized areas are cropped out of the raw images and used to build a dataset of single colony images containing either one of the four individual morphological classes (Dense, Differentiated, Spread, Debris) or multi-label images that contain more than one morphological class. The binary maps are also used as a boundary from which patches are taken within the borders of the cell colony, such that every image contains relevant class information. During training, random image patches are selected from the image within the colony ROI. An example of a binary map for a gray scale input image can be found in Fig. 5.

#### Multi-scale input

There is an important distinction to make when referring to scale and resolution in terms of optical microscopy and image properties. For the optical light microscope used to capture the image dataset in this work, the term resolution refers to the smallest distance between two discernible points of light captured within an image, whereas scale is used to determine the size of objects that can be observed within the image. For images captured using a specific objective magnification (in this case 10x), the resolution of the image is a fixed value that is related to the spacial distance represented by a given number of pixels, and scale is related to the number of pixels comprising an image in a given region-of-interest. These two terms can sometimes be used interchangeably when discussing images because they can both have similar effects on image output (i.e. changing image scale can also affect the resolution of the image). For the purposes of this paper, the term scale is taken to mean the size of the input image at a given resolution, and therefore multi-scale refers to taking multiple size patches from the original image with a fixed resolution.

Using multi-scale inputs can have several advantages over a single-scale because of the nature of deep feature extraction, including image down-sampling steps performed by the neural network which result in low-level feature representations of the input image, where some fine grained features can be lost in feature space. Conversely, when providing multiple image scales, features at multiple input levels can be provided to the network for training, which allows for modeling of local and global features separately. For example, Christiansen et al. perform in-silico labeling of histopathology images by using multi-scale inputs to convert immunohistopathological images to fluorescent staining without the use of fluorescent microscopy^38^. They successfully segment various cellular structures using a multi-head output, but their network is very large and extremely expensive computationally, making it impractical to train in a normal research setting. The importance of input feature variability cannot be over-emphasized when trying to improve model training. Both global and local features contribute to model learning, and multi-scale inputs can help to improve classification accuracy for deep learning models by providing multiple views of the input at different scales.

Given these considerations, the method in this paper leverages multi-scale, and multi-label inputs, and performs resampling of an imbalanced dataset using pseudo-labels for morphological image patches to improve feature extraction during training. This approach allows for colony images containing multiple classes to be used in a patch wise manner to inform model learning by increasing the features available to the network. It is shown empirically here that this method displays improvement over standard convolutional neural networks, as well as similar methods of contrastive learning for biological datasets.

**Figure 4:**
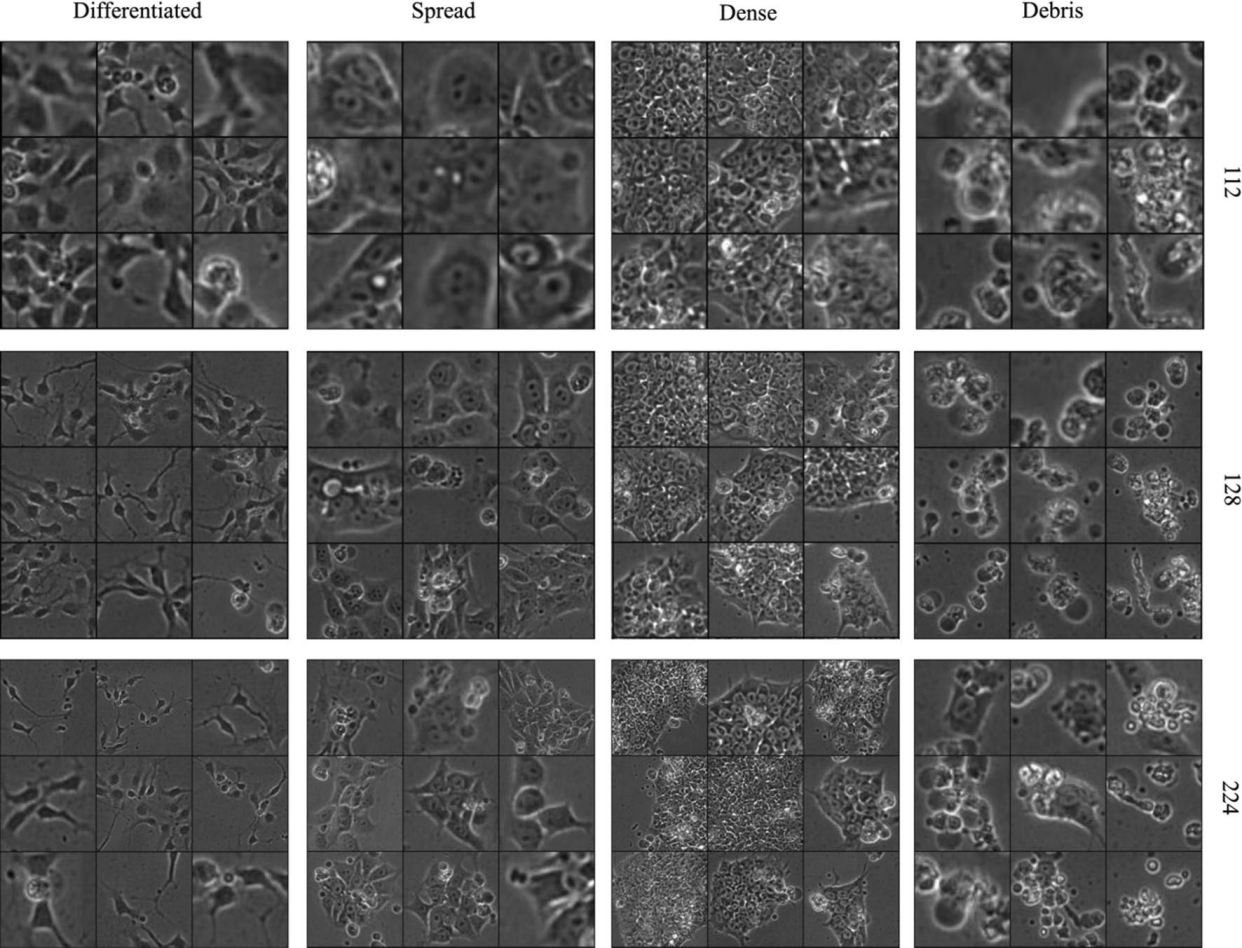
Sample image patches for every class at three scales (112, 128, and 224). Different views of image features are provided at each scale. At the lowest scale, 112 $\times$ 112, local views of fine-grain texture patterns are predominant. At the 128 $\times$ 128 scale, local texture features are present but global features, such as edges, are also observable. At the 224 $\times$ 224 scale colony shape becomes an important feature because images contain views of entire colonies.

The single-scale networks used for the training configurations in this work are modified to accept multi-scale inputs. For the single scale architecture, size 128 $\times$ 128 image patches are extracted from the training images as input for the network. For the multi-scale configuration, the high scale input is a 224 $\times$ 224 image patch and the lower scale is a 112 $\times$ 112 center patch of the higher scale image. This allows for the resulting feature vectors of the two inputs to be concatenated at equal lengths before the final classification layers of the network. A diagram of the multi-scale VGG network architecture is shown in Fig. 3, and image samples at various scales can be seen in Fig. 4.

These input scales are determined based on the relationship between optical properties of the microscope used for data collection and the relevant optical feature scale of cellular colonies in the images. Individual cell sizes can range from 10-100 $\mu m$, and due to the combination of optical parameters of the microscope unit used for data collection in this work, the individual pixel size of the live cell images is 0.8 $\mu m$^44^. Therefore, a line of 128 pixels equates to 102.4 $\mu m$ within the region of interest. The larger scale image patch, 224 $\times$ 224, encompasses a more global view of the input image (179.2 $\mu m^2$) and contains features such as colony shape, edges, and surrounding area. The smaller-scale input patch, 112 $\times$ 112 (89.6 $\mu m^2$), captures the morphological texture patterns of the cellular area within a colony. Both image patch scales contribute useful information for the network to learn, and the concatenation of learned feature vectors provides more information for the network to use in its classification decision.

The networks are then trained to learn features of the input patches and sort them into the four morphological classes. Some of the images contain areas of more than one image class and because they were captured without the use of stains of fluorescent biomolecules, they would not be usable in traditional deep learning training configurations which necessitate image level labels as a minimal requirement. However, using the IPB algorithm, image patches from multi-label images can be used to train the student network when given pseudo-labels by the teacher network.

#### Multi-label input

**Figure 5:**
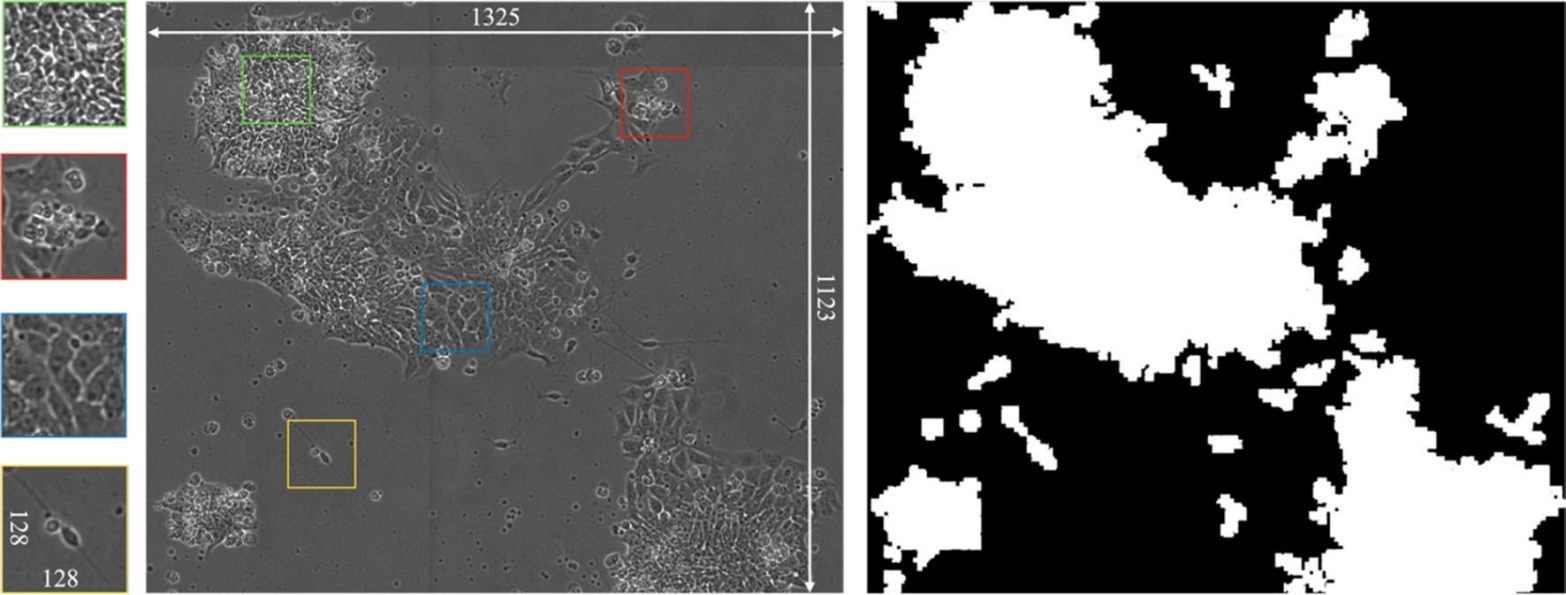
(Right) Binary map of cell colony area calculated using morphological segmentation. This map is used to reduce the presence of background area when taking image patches. (Center) Example of a large-scale (1325 $\times$ 1123) multi-label image containing areas of each of the individual four classes. (Left) 128 $\times$ 128 patches of images and their corresponding locations within the larger image (from top to bottom: Dense, Debris, Spread, Differentiated). The high background to foreground ratio makes taking random patches from within the binary map of the colony a crucial step in providing relevant information to the model.

Figure 5 provides an example of a multi-label colony image that contains areas of all four of the relevant image classes. The colonies in these images contain multiple cell classes with contiguous boundaries. As described above, it is very difficult to provide sub-image level labels for these inputs because there exists no pixel level ground-truth in the dataset and as a result these images are useless for training traditional CNN architectures using reinforcement learning. However, the IPB algorithm provides an avenue for including image patches from multi-label images without providing semantic labels.

This is accomplished by simply providing image patches of multi-label images to the teacher network and conserving those image patch areas for each minibatch of inputs. Additionally, the MPL algorithm determines both soft pseudo-labels, as well as hard pseudo-labels provided to the student network using a prediction confidence threshold of than 0.65. In this way, any class overlap that might be captured in a random image patch can be filtered out before reaching the student network. By including multi-label images, the network is provided with more variability of class input features. Random image patches are resampled for every epoch, allowing the network to learn from a balanced dataset distribution on the fly.

#### Dataset augmentations

Every random patch input is transformed using a series of random augmentations that includes horizontal and vertical flipping, 0-180 degree rotation, contrast and brightness adjustment, and gaussian blurring. Each of these augmentations is applied independently with a 0.25 probability, and they help to increase the effectiveness of the UDA module that is used to train the teacher network. Furthermore, several inherent augmentations are included in dataset pre-processing which also contribute to variability within the training dataset.

For example, patch crops provide an intrinsic randomized view of input images during each training epoch and introduce different morphological patterns and foreground to background ratios. Also, images that are too small to be cropped within the given patch size necessitate a resizing augmentation that helps account for scale invariance within the image data. These examples of innate augmentations highlight the relevance of biological variability in improving network generalization by expanding the apparent size and scope of the input dataset. Specifically those factors relating to the collection of microscopy data, which can encompass multiple scales, lighting levels, and optical parameters.

#### Iterative pseudo balancing resampling scheme

The pseudo label resampling scheme proposed in this work is a crucial step in balancing the input dataset for IPB learning. An overview of the pre-training, training, and testing steps are provided in previous sections. In line with the standard MPL algorithm, the pre-trained teacher provides pseudo labels to patch samples of the unlabeled training dataset. Where normally these pseudo labeled images would then be directly used to update the student network, IPB adds an intermediate step that involves balancing the pseudo-labeled image patches by weighting each class based on their relative probabilities within the dataset.

At the beginning of each epoch, the teacher network provides a pseudo label to each random image patch from the unlabeled dataset. Given a dataset with *m* classes, the minimum number of samples for a given class is $n_i = \min (n_1, n_2, \ldots , n_m)$ for $i = 1 \rightarrow m$. The proportion of every class in the unlabeled dataset ($p_i$) is determined using the pseudo-labels and samples are drawn from a multinomial distribution with replacement using the inversely proportional weights of the classes within the dataset. The number of images taken from a given class $n_i = p_i * n_i$ such that the total number of images taken from a given class can vary given the proportion of image labels in the dataset of image patches. The resampled dataset is used to update the student network, and the student networks performance on the labeled dataset is used to update the teacher networks such that it can provide more accurate pseudo-labels to the student.

Inefficiences in network learning caused by class imbalances are due to the effect of confirmation bias in model learning^45^, which is when the teacher network overfits on the most prevelent class, so that when the student is provided with random, pseudo-labeled data points, the proportion of these images coming from a given class cannot be controlled, and given the high levels of imbalance present in the dataset used for this work, the network begins to only learn features of the most prevalent class. The IPB resampling scheme proposed in this work overcomes the problem of confirmation bias due to class imbalance by using the pseudo-labels to balance the image dataset on the fly for every training iteration.

**Algorithm 1:**
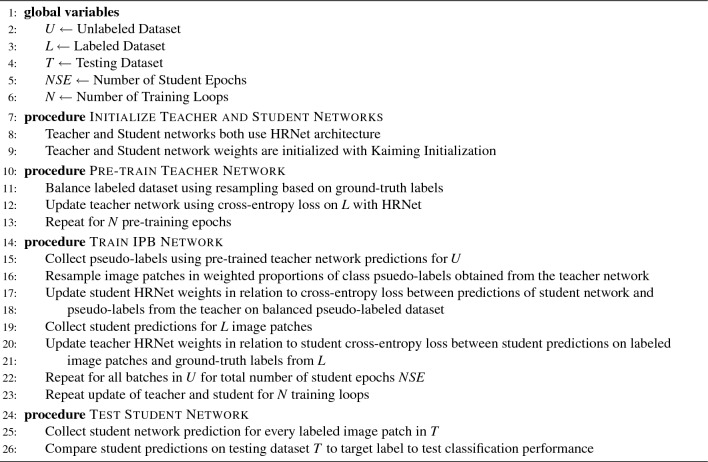
Iterative Psuedo Balancing Algorithm.

## Results and discussion

### Dataset and ground-truth

The input dataset for this work is composed of time-lapse, phase-contrast microscopy images of induced pluripotent stem cells. The goal of these experimental studies is to determine the developmental effects of nicotine on Huntington’s Disease affected neurogenesis. The study was designed and conducted by Dr. Barbara Davis from the Laboratory of Dr. Prue Talbot at the University of California, Riverside, Stem Cell Center. The experimental design centers around the premise that nicotine has a neuroprotective effect on cells affected by neurodegenerative diseases and involves culturing the cells under control and experimental conditions to observe their behavior over the course of 48 hours using the Nikon Biostation CT incubator/microscope unit. This microscope outputs 2908 $\times$ 2908 stitched images of the entire culture well at 10x optical magnification, taking one image every hour over the course of the experiment. In total, the dataset includes 15, 48 image time-lapse sequences, which are pre-processed and annotated before being used for network training.

Cropped colony ROI’s from the dataset of raw microscope images are sorted into four morphological classes that correspond to the unique phenotypes that are observed during the experiment. These classes include Dense, Spread, Differentiated, and Debris, each characterized by distinct texture and shape features that translate directly to the health and developmental status of the cell colonies. More specifically, the *Dense* class corresponds to early stage pluripotent stem cells, with relatively small individual size and dense, uniform, fine grained texture; the *Spread* class indicates intermediate stage progenitor cells with larger cell body area and a more uneven texture pattern; the *Differentiated* class represents adult neurons with dark cell bodies and elongated protrusions called axons; the *Debris* class displays spherical groups of dying or dead cells with high contrast indicative of the bubble like rounding of the dead cells which leads to shiny white areas around the perimeter.

The ground-truth labels were determined by a skilled biologist along with two other individuals who were trained to distinguish between the individual classes by visual appearance and provided with exemplary image references. Final ground-truth was determined via majority vote of annotations for each colony image. Table 1 details the breakdown of the number of images that fall into each data class. Images range a variety of scales, with the smallest being 55 $\times$ 85 pixels, and the largest being 771 $\times$ 1298. Colony images that contain areas with more than one morphological class label were sorted into a separate bin for multi-label images. These 2,659 images in the dataset were previously impractical to use for neural network training because their predicted class cannot be compared to any single ground-truth, however, patches of these images are given pseudo labels in conjunction with the rest of the dataset during IPB training. Subcategories of these images include partially differentiated colonies (1,436 images), that contain at least some portion of the differentiated class, as well as various proportions of the other downstream classes; and partially spread (1,223 images) which similarly contain some portion of spread class as well as various proportions of downstream classes. The training dataset contains both labeled and unlabeled images, where the majority of the dataset (80%) is used as unlabeled data for the semi-supervised IPB algorithm, a smaller portion (10%) is used for the supervised pre-training, fine-tuning and teacher update steps, and another small portion (10%) is used for model validation.

**Table 1:** Breakdown of data samples per class for the stem cell microscopy dataset.

Class	# Samples
Debris	3587
Dense	3934
Diff	656
Spread	10506
Multi-label	2659
Total	21342

True Positive Rate (TPR) and F1 score are used as metrics for classification accuracy because for an imbalanced dataset, TPR delineates how misclassifications affect the performance of the network, and F1 score computes the harmonic mean of precision and recall, providing a comprehensive view of the models susceptibility to false positives and false negatives. TPR and F1 score are calculated as as shown in Eqs. (3) and (4). The results of the training configurations containing these images is detailed in the following sections.

3 $$\begin{aligned}{} & {} TPR = \frac{TP}{TP + FN} \end{aligned}$$

4 $$\begin{aligned}{} & {} F1 = \frac{2TP}{2TP + FP + FN} \end{aligned}$$

### Pseudo-label resampling scheme accurately predicts class weights for training dataset

The first important step in training any of the experimental IPB configurations is the resampling scheme proposed in this work. Without performing dataset balancing via pseudo label resampling as outlined in the previous section, the MPL network overfits on the most prevalent class, resulting in imbalanced classification. However, by adding this step, the student network learns normally from a balanced dataset after the teacher performs pseudo-labeling.

**Figure 6:**
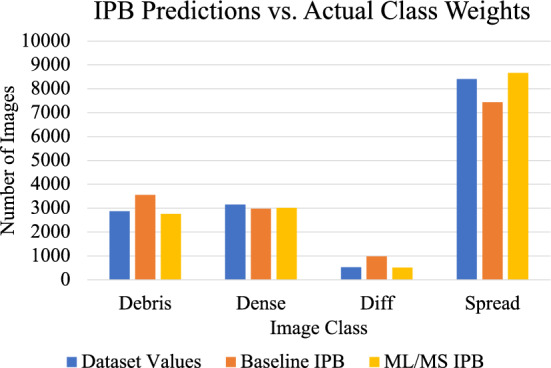
Predicted class weights from the teacher network for two IPB configurations, as well as the actual training dataset distribution. The multi-scale input patches from multi-label images (ML+MS) allows the teacher to learn a more accurate distribution of the image classes, which it can then use to provide the student with a more accurate pseudo-balanced dataset.

**Figure 7:**
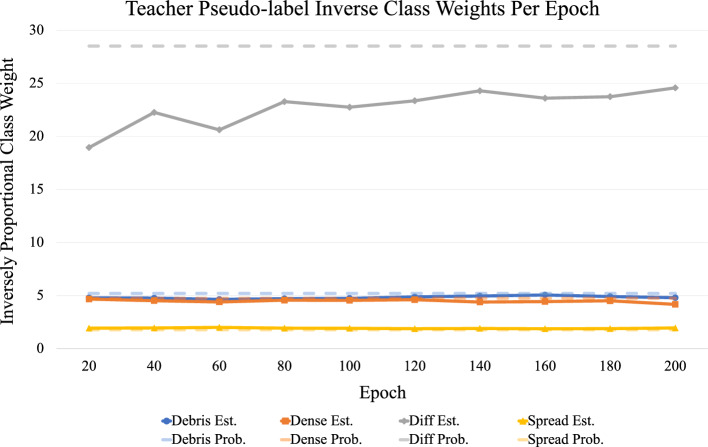
Sampling weights for each class displayed as the inverse proportion of pseudo-labels as predicted by the teacher network across the unlabeled image dataset.

This is important because one of the main limitations of semi-supervised networks is the inability to learn effectively on imbalanced datasets. Furthermore, biological datasets are often highly imbalanced, so the psuedo-label resampling scheme allows for the unlabeled images to contribute equally to the multi-modal distribution learned by the neural network. This is illustrated in Fig. 6, which displays the predicted class proportions of various networks. The proportions are determined by accumulating the teacher networks predictions across the labeled training dataset, which are then used to balance the dataset of image patches during each training epoch. Figure 7 shows the teachers progression in estimating the proportions of every class in the unlabeled dataset by providing each image a pseudo-label. As training progresses, the teacher begins to learn a more balanced distribution of the dataset, as evident by the values for the differentiated class trending towards the actual class proportion.

### Ablation experiment: multi-scale and multi-label inputs provide global features without the need for annotation

The multi-scale IPB configurations (MS HRNet, MS IPB VGG, MS IPB HRNet) which take both 224 and 112 scale inputs to create a combined feature vector, show the greatest improvement over the baseline architectures, in the Dense and Differentiated classes, especially for the HRNet model configuration. These observations may be due to the global features that are incorporated into feature learning with the multi-scale input. Specifically, the introduction of morphological features such as edges, colony shape and size, and foreground to background contrast contribute to improved feature discrimination during model learning. This includes a statistically significant increases in the TPR of the Dense class by $\sim 5\%$ (P-value: 0.0009 using student t-test with 95% confidence value) and the Differentiated class by $\sim 7\%$ (P-value 0.0448 using student t-test with 95% confidence) when compared to the baseline HRNet. For the other classes, these features are already present or prominent in the single-scale input or do not contribute additional information at a higher-scale (Fig. 4).

The multi-label IPB configurations (ML IPB VGG, ML IPB HRNet) includes single scale input patches of unlabeled training images that contain multiple image classes within a single colony. By taking random crops of these images iteratively during training, the number of additional unique features that these images contribute is extremely large. These improvements in model learning can be attributed to the increase in available data-points for feature extraction. Another possible consideration for the multi-label images is that they represent some later stage cell colonies because of the larger colony size and presence of multiple classes. This could account for some of the improvement in the Dense class as well as classes representing the later stage phenotypes (Differentiated and Spread).

### Combining multi-label and multi-scale inputs maximizes available features for model learning

While the separate implementation of multi-label patches and multi-scale inputs results in significant improvement over baseline CNN and IPB implementations, the combination of these modules for IPB training demonstrates the true power of these features when used in conjunction. Tables 2 and 3 contain the classification results for the IPB training configurations in relation to a standard VGG and HRNet CNN architectures in terms of TPR and F1 Score.

**Table 2:** True positive rate of classification for all IPB configurations, where Multi-scale is denoted as MS, and Multi-label is denoted as ML.

Config./Class (std.)	Debris	Dense	Diff	Spread	Average
VGG19	0.8877 (0.0174)	0.7772 (0.0308)	0.8434 (0.0396)	**0.9299 (0.0134)**	0.8595
HRNet	0.8670 (0.0087)	0.7752 (0.0291)	0.8162 (0.0569)	0.9239 (0.0076)	0.8455
MS HRNet	0.7921 (0.0491)	0.8410 (0.0116)	0.9013 (0.0595)	0.8813 (0.0111)	0.8539
IPB VGG	0.8389 (0.0615)	0.7695 (0.0590)	0.7971 (0.0621)	0.9281 (0.0277)	0.8334
MS IPB VGG	0.8843 (0.0576)	0.8489 (0.0344)	0.8369 (0.0913)	0.8949 (0.0340)	0.8662
ML IPB VGG	0.8890 (0.0356)	0.8061 (0.0467)	0.8516 (0.0605)	0.9144 (0.0190)	0.8647
ML+MS IPB VGG	0.8905 (0.0419)	0.8529 (0.0312)	0.8259 (0.0741)	0.9006 (0.0148)	0.8674
MS IPB HRNet	0.8934 (0.0512)	0.8241 (0.0295)	0.8846 (0.0826)	0.9112 (0.0174)	0.8783
ML IPB HRNet	**0.9139 (0.0364)**	0.7920 (0.0311)	0.8737 (0.0785)	0.9139 (0.0158)	0.8733
ML+MS IPB HRNet	0.8843 (0.0159)	**0.8575 (0.0218)**	**0.9085 (0.0189)**	0.8985 (0.0074)	**0.8872**

Bold values represent best result for each column.

**Table 3:** F1 Score for all IPB configurations, where Multi-scale is denoted as MS, and Multi-label is denoted as ML.

Config./Class (std.)	Debris	Dense	Diff	Spread	Average
VGG19	**0.8723 (0.0161)**	0.8342 (0.0162)	0.8460 (0.0255)	0.9053 (0.0073)	0.8644
HRNet	0.8579 (0.0111)	0.8215 (0.0154)	0.8565 (0.0174)	0.9011 (0.0055)	0.8567
MS HRNet	0.8122 (0.0206)	0.8071 (0.0066)	0.7628 (0.0541)	0.8901 (0.0072)	0.8180
IPB VGG	0.8497 (0.0245)	0.8152 (0.0142)	0.8037 (0.0678)	0.8899 (0.0143)	0.8396
MS IPB VGG	0.8529 (0.0374)	0.8339 (0.0275)	0.8190 (0.0439)	0.9044 (0.0109)	0.8525
ML IPB VGG	0.8627 (0.0228)	0.8293 (0.0189)	0.8519 (0.0300)	0.9063 (0.0060)	0.8625
ML+MS IPB VGG	0.8574 (0.0182)	**0.8513 (0.0196)**	0.8204 (0.0463)	0.9102 (0.0104)	0.8598
MS IPB HRNet	0.8608 (0.0141)	0.8412 (0.0158)	0.8536 (0.0346)	0.9125 (0.0036)	0.8670
ML IPB HRNet	0.8580 (0.0146)	0.8419 (0.0188)	0.8587 (0.0486)	0.9067 (0.0057)	0.8663
ML+MS IPB HRNet	0.8609 (0.0102)	0.8422 (0.0020)	**0.8749 (0.0278)**	**0.9132 (0.0030)**	**0.8728**

Bold values represent best result for each column.

Overall, the ML+MS IPB HRNet improves classification accuracy as measured by TPR over the standard CNN architectures by $\sim$3%, with statistically significant improvements in the Dense ($\sim 8\%$), and Differentiated ($\sim 9\%$) Classes using a student t-test with a 95% confidence rate (p-values: 0.0001, and 0.0002, respectively). As previously stated, the introduction of global features using multi-scale inputs improves feature disentanglement by providing context for the networks class prediction. Additionally, these improvements suggests that pseudo-balancing the multi-label and multi-scale data allows the network to learn from a more balanced distribution of class features.

In terms of F1 score, the ML+MS IPB HRnet configuration also shows the greatest improvement over the baseline CNN, by $\sim$2%, with statistically significant improvements in the Dense class via student t-test (p-value: 0.0005). These improvements may be due to the fact that HRNet has the capacity to conserve high-level features such as shape characteristics of the larger scale input as well as model texture features present at the smaller scale input patches.

These improvements can be attributed to the interplay between the multi-label and multi-scale image samples that are present in the dataset. For example, the 2,659 additional images that are introduced for the multi-label configuration tend to represent larger colonies from later-stage images. When taking single scale patch samples of these images, the surrounding area is not taken into consideration, which results in fewer available features for the network. Conversely, the global information provided by the multi-scale input may not provide as much additional information for model learning when applied to the smaller, single-class images. However, when combined, the multi-scale, multi-class input provides useful features from a large portion of the dataset that was previously unusable in a supervised network setting.

### Reducing proportion of labeled training data for data limited settings lowers classification performance

To test the ability of the model to learn on smaller proportions of labeled data for use in the teacher pre-training and MPL update steps, network configurations were trained using 1%, and 5% as well as 50% and 80% of labeled data and compared to the standard 10% configuration used in this work. The lowest data setting (1%) represents only 191 total labeled images, whereas the 5% setting contains 938 labeled images, the 10% setting contains 1,873 labeled images, the 50% configuration contains 9,341 labeled images and the 80% configuration contains 14,946 labeled images.

**Figure 8:**
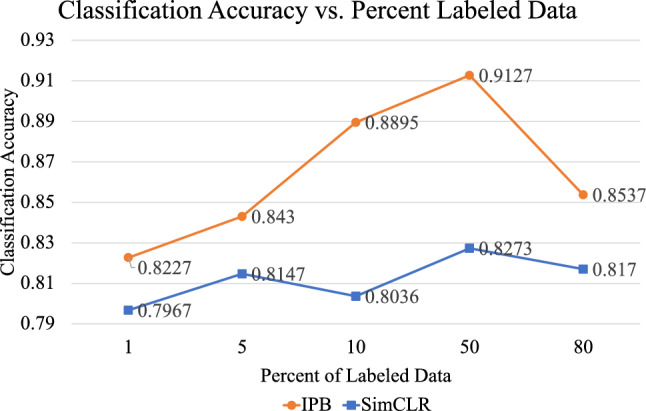
Graph of classification accuracy vs. proportion of labeled data used for training. There is a positive correlation between the amount of labeled data and the final classification accuracy of the student. However, these results still demonstrate that in a data limited setting, this method can still be effective.

Figure 8 demonstrates a general upward trend in classification accuracy for the IPB network configuration until the 50% data mark, when for the 80% configuration, the accuracy of the model declines. This is because the student network is trained on images with pseudo-labels from the teacher network, which become more robust with increased number of labeled data points until the amount of data used to generate confident pseudo-labels results in a lack of sufficient data points with which to train the student network effectively. However, the results using the lower data settings still highlight the potential of this method to train the neural network even in very data limited settings.

These dataset configurations are also used to train the SimCLR model, which is the most competitive related work to the proposed method. This self-supervised method has the limitations of not being able to estimate the dataset distribution, as well as requiring a large amount of data for effective feature mapping. This can be seen in Fig. 8 where fluctuations in accuracy can be seen, with a general upward trend. Given the size of the dataset used in this work, there are not enough data points for the network to learn effectively, resulting in overall lower performance as compared to the IPB method proposed in this work, which obtains superior results to this method using only a fraction of the training data.

### Observation of misclassifications highlights importance of multi-scale network

**Figure 9:**
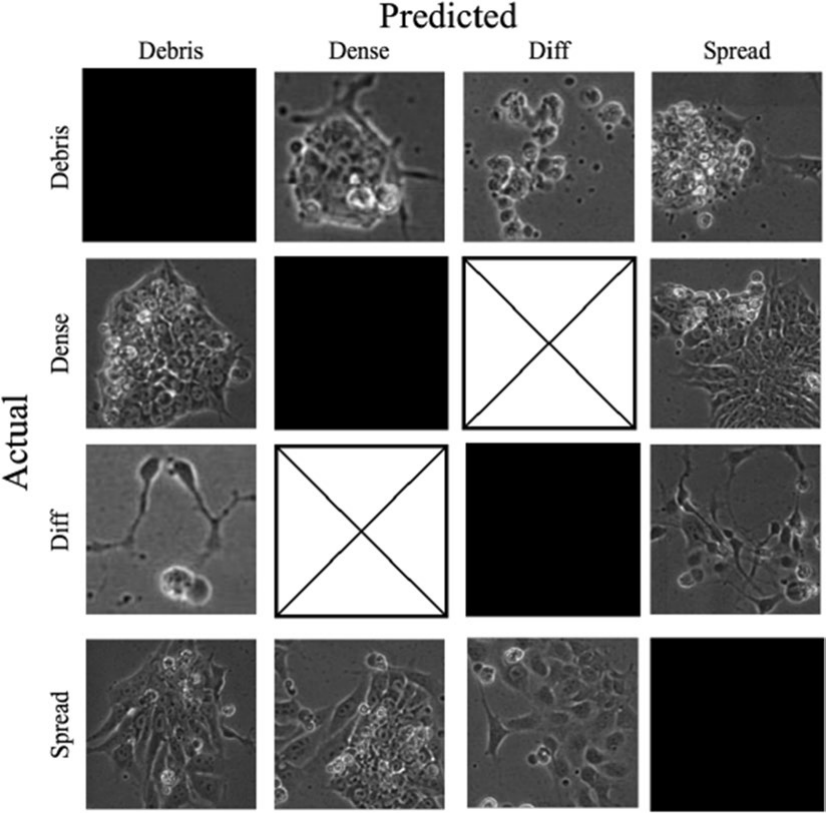
Example misclassifications for the multi-scale/multi-label configuration. Black boxes represent the omitted correct classifications, and boxes with and x through them represent instances where no misclassifications occurred. Figure 4 provides example images of each of the classes.

Figure 9 shows examples of misclassified patches for the multi-label/multi-scale configuration. Several observations can be made including that misclassifications as Debris are often due to the presence of dead or dying cells within or surrounding healthy colony areas; Spread images misclassified as Dense sometimes contain areas with more than one cell class present and vice versa for the Dense to Spread class; misclassified differentiated images are confused by the network because they can appear flat like a spread image, but no differentiated images were improperly predicted as Dense and vice versa, mostly because these classes are the least closely associated with one another biologically, whereas the Spread class is the intermediate between the Dense and Differentiated, and is the most prevalent and most commonly confused class. Table 4 portrays a representative confusion matrix for these network predictions, which highlights the misclassifications caused by the presence of feature overlap between biologically adjacent classes. These observations help to emphasize the importance of incorporating a multi-scale input, which can provide a view of both the fine grained textures, and global feature patterns of the input images.

**Table 4:** Representative confusion matrix for the IPB network.

Predicted/Actual Class	Debris	Dense	Diff.	Spread
Debris	301	18	2	37
Dense	11	326	0	56
Diff.	1	0	62	2
Spread	32	39	6	973

The most confused classes observed here are the Dense, Debris, and Spread classes, which can be attributed to the presence of class overlap as a result of downstream differentiation. Similarly, there are no misclassifications between the Dense and Differentiated classes because of their relatively far distance in terms of biological proximity.

### Multi-scale pseudo-balancing network out-performs state-of-the-art

Several previous works have attempted to perform semi-supervised and self-supervised learning for natural image and biological datasets. To determine the effectiveness of these methods in relation to the proposed approach, four state-of-the-art models (MPL^33^, SimCLR^25^, Contrastive Clustering^27^, and SimTriplet^30^) were trained on the dataset, and the results were calculated across 5 folds in Table 5. These results confirm that the major pitfall of these methods is that they fail to account for class imbalances in the absence of prior information. Furthermore, contrastive methods such as SimCLR work most effectively with very large amounts of data, which is counterintuitive to the tasks associated with biological image datasets that are inherently limited by the constraints of experimental research.

**Table 5:** True Positive Rate for related works in comparison to the multi-label, multi-scale IPB configuration, where Multi-scale is denoted as MS, and Multi-label is denoted as ML.

Config./Class (std.)	Debris	Dense	Diff	Spread	Avg.
SimCLR^25^	0.8559 (0.0346)	**0.8712 (0.0274)**	0.7538 (0.0592)	0.7874 (0.0213)	0.8170
CC^27^	0.5101 (0.1756)	0.3852 (0.4324)	0.2277 (0.2370)	0.3436 (0.0841)	0.3666
SimTriplet^30^	0.1726 (0.0266)	0.7160 (0.1390)	0.2246 (0.1864)	0.5423 (0.1012)	0.4138
MPL (Imbalanced)^33^	0.8158 (0.0804)	0.8423 (0.0407)	0.8818 (0.0504)	**0.9292 (0.0237)**	0.8672
MPL (Balanced)	**0.8950 (0.0106)**	0.8261 (0.0540)	0.8322 (0.0564)	0.9111 (0.0221)	0.8661
IPB (ML+MS)	0.8843 (0.0159)	0.8575 (0.0218)	**0.9085 (0.0189)**	0.8985 (0.0074)	**0.8872**

Related methods display inferior results due to the effect of class imbalances, which causes over-fitting and high variance.

Bold values represent best result for each column.

The MPL algorithm does not inherently account for class imbalances, and also tends to bias towards on the most prevalent class (Spread). The MPL algorithm trained with the pseudo-balancing module (MPL Balanced, Table 5) has a lower variance in interclass classification accuracy in comparison to the MPL Imbalanced configuration (0.0018 vs. 0.0024), which means that the results provide a more balanced classification performance. Furthermore, these MPL configurations show inferior performance to the IPB algorithm, which incorprates multi-scale and multi-label image patches to improve feature extraction and classification. The IPB algorithm outperforms the MPL configuration by 2% by providing the model with a balanced view of the input classes and as well as a global view of input data and incorporates image features from multi-label image patches. It also does not necessitate the large amount of data required for learning image features in a fully supervised manner because it utilizes semi-supervised pseudo-labeling to guide mapping of the input distribution.

## Conclusions

The approach presented in this work, Iterative Pseudo Balancing, represents a significant improvement in semi-supervised methods for resourceful management of biological datasets. Pseudo-labels from an MPL framework are used to iteratively balance a biological image dataset on the fly, and simultaneously train a student-teacher ensemble network to accurately classify stem cell colonies. Previously unusable, multi-label images are used to increase the available data points for model learning, and multi-scale inputs are used to integrate global and local features present in the dataset. Combining these views of the input data for an HRNet results in an overall improvement of 3% over the baseline CNN in terms of TPR, which is shown to be statistically significant. The results shown in this paper highlight the importance of balanced learning in biological experimentation when employing deep neural networks.

The IPB framework proposed here overcomes the problem of confirmation bias via overfitting as a factor of dataset imbalance by providing the network with a comprehensive view of the dataset, incorporating multiple image scales and exposing hidden features from mutli-label images. The exhaustive use of all available data and domain information is crucial to improving model performance when training on biological datasets. This work allows for biological researchers to easily employ deep learning analysis to their experimental datasets without having to spend time manually labeling large datasets, and to model their data using non-invasive morphological classification without the need for molecular biomarkers. In turn, this will accelerate the pace of biological research involving predictive models. Other possible avenues of exploration for improving this work include adding motion information for dynamics, and the collection of biomarker data for endpoint ground-truth.

## Data Availability

The dataset analyzed is available from the corresponding author on reasonable request. The code developed here is available via Zenodo^46^.
